# Purification and Characterisation of Malate Dehydrogenase From *Synechocystis* sp. PCC 6803: Biochemical Barrier of the Oxidative Tricarboxylic Acid Cycle

**DOI:** 10.3389/fpls.2018.00947

**Published:** 2018-07-13

**Authors:** Masahiro Takeya, Shoki Ito, Haruna Sukigara, Takashi Osanai

**Affiliations:** School of Agriculture, Meiji University, Tokyo, Japan

**Keywords:** biochemistry, cyanobacteria, malate dehydrogenase, metabolic enzyme, TCA cycle

## Abstract

Cyanobacteria possess an atypical tricarboxylic acid (TCA) cycle with various bypasses. Previous studies have suggested that a cyclic flow through the TCA cycle is not essential for cyanobacteria under normal growth conditions. The cyanobacterial TCA cycle is, thus, different from that in other bacteria, and the biochemical properties of enzymes in this TCA cycle are less understood. In this study, we reveal the biochemical characteristics of malate dehydrogenase (MDH) from *Synechocystis* sp. PCC 6803 MDH (*Sy*MDH). The optimal temperature of *Sy*MDH activity was 45–50°C and *Sy*MDH was more thermostable than MDHs from other mesophilic microorganisms. The optimal pH of *Sy*MDH varied with the direction of the reaction: pH 8.0 for the oxidative reaction and pH 6.5 for the reductive reaction. The reductive reaction catalysed by *Sy*MDH was activated by magnesium ions and fumarate, indicating that *Sy*MDH is regulated by a positive feedback mechanism. The *K*_m_-value of *Sy*MDH for malate was approximately 210-fold higher than that for oxaloacetate and the *K*_m_-value for NAD^+^ was approximately 19-fold higher than that for NADH. The catalytic efficiency of *Sy*MDH for the reductive reaction, deduced from *k*_cat_-values, was also higher than that for the oxidative reaction. These results indicate that *Sy*MDH is more efficient in the reductive reaction in the TCA cycle, and it plays key roles in determining the direction of the TCA cycle in this cyanobacterium.

## Introduction

Cyanobacteria performing oxygenic photosynthesis synthesise various compounds from carbon dioxide using light energy. Cyanobacteria are widely used as hosts in metabolic engineering to produce renewable resources. *Synechocystis* sp. PCC 6803 (hereafter *Synechocystis* 6803) is one of the most highly studied cyanobacteria because it has many advantageous features, such as rapid proliferation and ease of transformation. Besides genetics, biochemical analyses of enzymes related to oxaloacetate metabolism proceed using *Synechocystis* 6803 enzymes (Ito et al., 2017; Takeya et al., 2017), and thus this cyanobacterium is widely used for basic studies of primary carbon metabolism.

The tricarboxylic acid (TCA) cycle is one of the most important biochemical reactions in aerobic energy production, and is common among most respiring organisms. Reductants are generated by oxidation of metabolites through the TCA cycle, leading to ATP production through the process of respiration, which uses these reductants. Metabolites in the TCA cycle, such as oxaloacetate and 2-oxoglutarate, are precursors of various metabolites, including amino acids, sugars, and lipids (Owen et al., 2002). The cyanobacterial TCA cycle is also involved in various metabolic systems, which can lead to the production of useful materials, such as succinate (Osanai et al., 2015), amino acids (Matsunaga et al., 1991), ethylene (Xiong et al., 2015) via acetyl-CoA, and TCA cycle derivatives from fixing carbon dioxide by oxygenic photosynthesis using light energy.

Compared to studies on enzymes in the Calvin cycle, biochemical analysis of enzymes of the TCA cycle in cyanobacteria is limited. The cyanobacterial TCA cycle was once thought to be an incomplete cycle owing to the lack of 2-oxoglutarate dehydrogenase (OGDH); however, it has been demonstrated that 2-oxoglutarate decarboxylase and succinate semialdehyde dehydrogenase produce succinate from 2-oxoglutarate (Zhang and Bryant, 2011; Steinhauser et al., 2012). In addition, the γ-aminobutyric acid (GABA) shunt produces succinate from glutamate in *Synechocystis* 6803 (Xiong et al., 2014), and the glyoxylic acid shunt is found in the cyanobacterium *Chlorogloeopsis fritschii* strain PCC 9212 (Zhang and Bryant, 2015). Thus, the cyanobacterial TCA cycles are potentially closed with these alternative shunts. However, these studies only analysed the first half of the TCA cycle, from citrate to succinate. The latter half of the TCA cycle has been studied by *in silico* analysis (Knoop et al., 2013; Rubin et al., 2015). Kinetic values, such as *k*_cat_ and *K*_m_, of cyanobacterial TCA cycle enzymes have not been determined, except for isocitrate dehydrogenase (Muro-Pastor and Florencio, 1992, 1994). Biochemical analysis of phosphoenolpyruvate carboxylase (PEPC), which produces oxaloacetate from phosphoenolpyruvate, reveals that *Synechocystis* 6803 PEPC is uniquely tolerant to feedback inhibition by malate and aspartate (Takeya et al., 2017). In addition to the oxidative cycle, the cyanobacterial TCA cycle reverses to a reductive reaction (called the reductive branch of the TCA cycle) under dark, anaerobic conditions (Hasunuma et al., 2016).

Malate dehydrogenase (MDH) is an enzyme that catalyses the interconversion between malate and oxaloacetate using NAD(P)H. MDHs are largely conserved in most species, irrespective of variation in the TCA cycle (Huynen et al., 1999; Minárik et al., 2002). MDH catalyses the oxidative reaction in the TCA cycle (malate to oxaloacetate) *in vivo*, although MDH thermodynamically prefers the reductive reaction (oxaloacetate to malate) *in vitro* (Molenaar et al., 1998). Thus, MDH is a unique enzyme that prefers the reductive reaction in the TCA cycle; however, the biochemical parameters of *Synechocystis* 6803 MDH (*Sy*MDH) have not been determined. MDH functions to protect against oxidative stress in *Escherichia coli* (Wu et al., 2007; Singh et al., 2008), also suggesting the physiological importance of MDHs in bacteria. In this study, *Sy*MDH was purified, and its biochemical functions were demonstrated for the first time, revealing unique regulatory mechanisms of *Sy*MDH.

## Materials and Methods

### Construction of Cloning Vectors for Recombinant Protein Expression

A *Bam*HI-*Xho*I DNA fragment of the *citH* (sll0891) ORF from the *Synechocystis* 6803 genome was amplified by PCR using KOD Plus Neo polymerase (Toyobo, Osaka, Japan) with the primers: forward, GAAGGTCGTGGGATCATGAATATTTTGGAGTATGCTC and reverse, GATGCGGCCGCTCGAGTTAACCGTCGCTAACCAT. The resultant fragments were excised with *Bam*HI-*Xho*I (Takara Bio, Shiga Japan) and cloned into the *Bam*HI-*Xho*I site of pGEX5X-1 (GE Healthcare Japan, Tokyo, Japan) using the In-Fusion HD Cloning Kit (Takara Bio, Shiga, Japan). Sequence integrity was confirmed by sequencing.

### Affinity Purification of Recombinant Proteins

Expression vectors were transformed into *E. coli* BL21 (DH5α, Takara Bio). Two litres of *E. coli* containing the vectors were cultivated at 30°C with shaking (150 rpm), and protein expression was induced overnight by adding 0.01 mM isopropyl β-D-1-thiogalactopyranoside (Wako Chemicals, Osaka, Japan).

Affinity chromatography was performed for protein purification as described in a previous study (Osanai et al., 2009). Two litres of *E. coli* cell culture were disrupted by sonication VC-750 (EYELA, Tokyo, Japan) for 5 min with 30% intensity and centrifuged at 5,800 ×*g* for 2 min at 4°C. The supernatant was transferred to a new 50-mL plastic tube on ice and 640 μL of glutathione-Sepharose 4B resin (GE Healthcare Japan) was mixed into the supernatant. After gentle rotating for 30 min, 1 mM ATP and 1 mM MgSO_4_⋅7H_2_O were added and samples were incubated with gentle shaking for 30 min to remove intracellular chaperons. After centrifugation (5,800 ×*g* for 2 min at 4°C), the supernatant was removed and resins were re-suspended in 700 μL of PBS-T (1.37 M NaCl, 27 mM KCl, 81 mM Na_2_HPO_4_⋅12H_2_O, 14.7 mM KH_2_PO_4_, 0.05% Tween-20) with 1 mM ATP/1 mM MgSO_4_⋅7H_2_O. The resin was washed with 500 μL of PBS-T (1.37 M NaCl, 27 mM KCl, 81 mM Na_2_HPO_4_⋅12H_2_O, 14.7 mM KH_2_PO_4_, 0.05% Tween-20) and eluted three times with 500 μL of GST elution buffer (50 mM Tris–HCl, pH 8.0, 10 mM reduced glutathione). Proteins were concentrated with VivaSpin 500 MWCO 50,000 spin columns (Sartorius, Göttingen, Germany) and protein concentration was measured with a PIERCE BCA Protein Assay Kit (Thermo Fisher Scientific, Rockford, IL, United States). Protein purification was validated by SDS-PAGE, including staining using InstantBlue (Expedion Protein Solutions, San Diego, CA, United States).

### Enzyme Assays

7.8 μg or 10 μg of *Sy*MDHs were used to measure oxidative or reductive reactions, respectively. The purified protein was mixed with 1 mL of assay solution (100 mM potassium phosphate buffer [pH 8.0 or pH 6.5], 0.1–32 mM nicotinamide adenine dinucleotide (NAD^+^), 0.01–0.64 mM nicotinamide adenine dinucleotide hydride (NADH), 0.2–32 mM malate, 0.02–0.4 mM oxaloacetate). The optimal temperature and the optimal pH were measured at the concentration exhibiting maximum activity (NAD^+^: 8.0 mM, NADH: 0.1 mM, malate: 4.0 mM, oxaloacetate: 0.1 mM). For the cell extract assay, cells from 1 L culture were collected by centrifugation and resuspend in 100 mM potassium phosphate buffer (pH7.0). The cells were disrupted by sonication and centrifuged at 5,800 × *g* for 30 min at 4°C. The protein concentration was quantified with BCA Protein Assay Kit (Thermo) and 420 μg of total proteins was added to 1 mL assay solution. Absorbance was measured at 340 nm using a UV-1850 spectrophotometer (Shimadzu, Tokyo, Japan). *V*_max_ and *K*_m_-values were determined using a Lineweaver–Burk double reciprocal plot. Results were plotted as a graph of the rate of reaction against the concentration of substrate and coenzyme using Kaleida Graph ver. 4.5 software. When the data did not show substrate inhibition, we performed curve fitting used the Michaelis–Menten equation (Eq. 1). When the data exhibited substrate inhibition, we performed curve fitting using the modified Michaelis–Menten equation (Eq. 2) (Eszes et al., 1996).

1 $$v=V\max[S]/([S]+K_{m})$$

2 $$v=V\max[S]/([S]+K_{m}+{\left[S\right]}^{2}/K_{\text{i}})$$

*v* and *V*_max_ indicate reaction velocity and maximum reaction velocity, respectively. [S], *K*_m_, and *K*_i_ indicate substrate concentration, the half-maximum concentration giving rise to 50% *V*_max_ and an inhibition constant, respectively.

## Results

### Measurement of Kinetic Parameters

**FIGURE 1:**
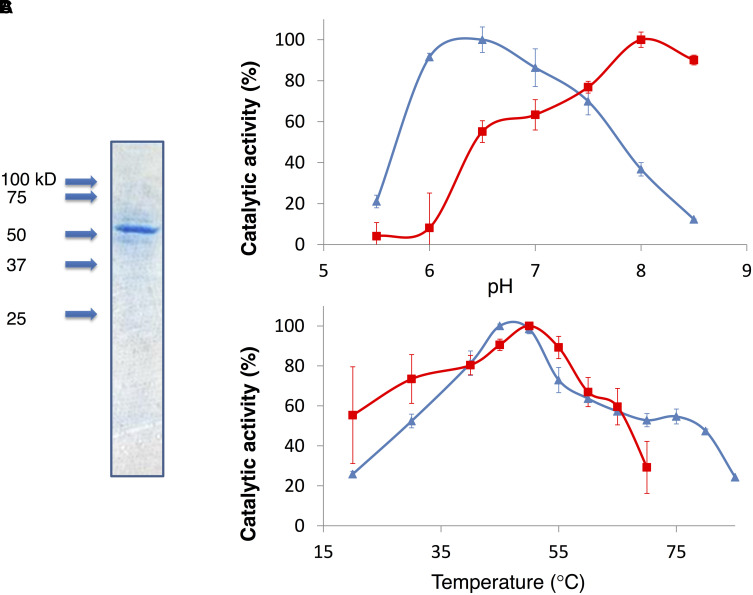
**(A)** Purification of GST-tagged *Sy*MDH. Proteins were electrophoresed on a 12% SDS-PAGE gel. The gel was stained with InstantBlue. Arrowheads indicate the molecular weight. **(B)** The effect of pH on *Sy*MDH activity. Red square represents the specific activity in the oxidative reaction (malate to oxaloacetate). Blue triangle represents the specific activity in the reductive reaction (oxaloacetate to malate). Data represent the relative values of the mean from three independent experiments. **(C)** The effect of temperature on *Sy*MDH activity. Red square represents the specific activity in the oxidative reaction (malate to oxaloacetate). Blue triangle represents the specific activity in the reductive reaction (oxaloacetate to malate). Data represent the relative values of the mean from three independent experiments.

**FIGURE 2:**
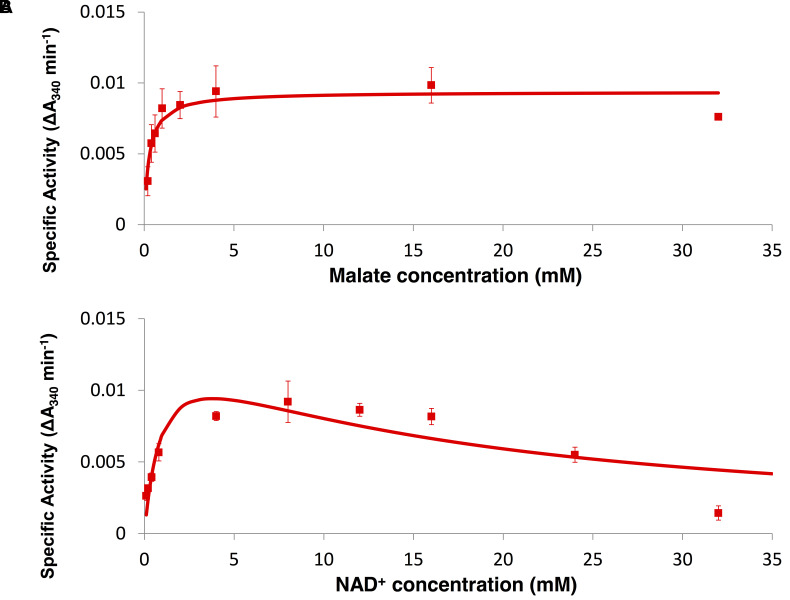
Enzyme assay of *Sy*MDH in the oxidative reaction *in vitro*. **(A)** Activity was measured by varying the malate concentration at a fixed NAD^+^ concentration (8.0 mM). The graphs show the mean ± SD obtained from three independent experiments. **(B)** Activity was measured by varying the NAD^+^ concentration at a fixed malate concentration (4.0 mM). The graphs show the mean ± SD obtained from three independent experiments.

**FIGURE 3:**
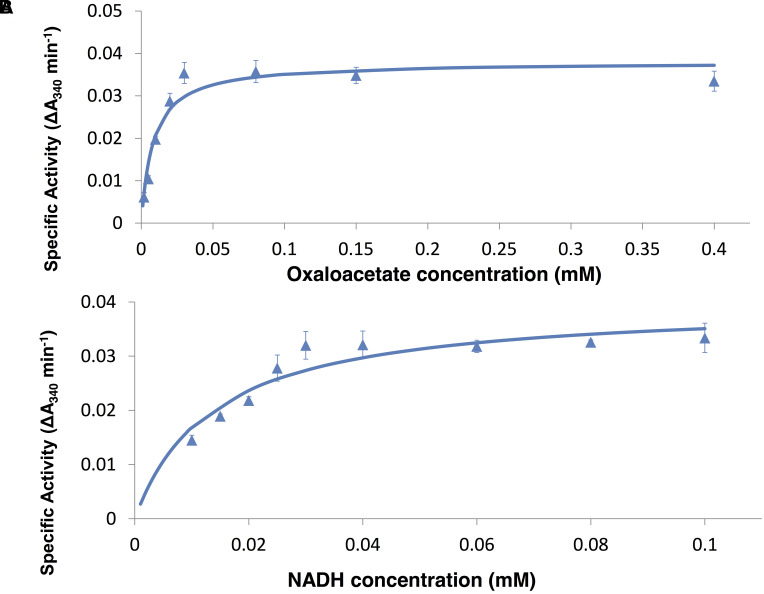
Enzyme assay of *Sy*MDH in the reductive reaction *in vitro*. **(A)** Activity was measured by varying the oxaloacetate concentration at a fixed NADH concentration (0.1 mM). The graphs show the mean ± SD obtained from three independent experiments. **(B)** Activity was measured by varying the NADH concentration at a fixed oxaloacetate concentration (0.1 mM). The graphs show the mean ± SD obtained from three independent experiments.

To determine the kinetic parameters of *Sy*MDH, glutathione *S*-transferase (GST)-tagged *Sy*MDH (GST-*Sy*MDH) proteins were expressed in *E. coli* and purified by affinity chromatography (**Figure 1A**). *Sy*MDH activity in the oxidative reaction (malate to oxaloacetate) was the highest at pH 8.0 and at a temperature of 50°C (**Figures 1B,C**). *Sy*MDH activity in the reductive reaction (oxaloacetate to malate) was the highest at pH 6.5 and at 45°C (**Figures 1B,C**). Kinetic parameters of *Sy*MDH were determined by a Lineweaver–Burk double reciprocal plot using the specific activity values in **Figures 2**, **3**. These results are summarised in **Tables 1**, **2**. *Sy*MDH displayed approximately 1.7-fold (*k*_cat_) and 361-fold (*k*_cat_/*K_m_*) preferences for oxaloacetate reduction over malate oxidation and approximately 4.7-fold (*k*_cat_) and 90.5-fold (*k*_cat_/*K_m_*) preferences for NADH oxidation over NAD^+^ reduction (**Table 1**). The catalytic efficiency of the reductive reaction was higher than that of the oxidation reaction for both the substrate and the coenzyme. The *K*_m_-value for malate was approximately 210-fold higher than that for oxaloacetate, and the *K*_m_-value for NAD^+^ was approximately 19-fold higher than that for NADH (**Table 2**). *Sy*MDH appeared to prefer oxaloacetate and NADH as substrate and coenzyme, respectively, *in vitro*. *Sy*MDH had enzymatic activity toward NAD^+^ and NADH, but no activity toward NADP^+^ and NADPH both *in vitro* and *in vivo* (Supplementary Figures S1, S2). We also determined kinetic parameters of *Sy*MDH using the Michaelis–Menten equation. These results are summarised in Supplementary Tables S1, S2. These calculations showed that *Sy*MDH prefers oxaloacetate and NADH as substrate and coenzyme, respectively; the *K*_m_-value for malate was approximately 84.4-fold higher than that for oxaloacetate, and the *K*_m_-value for NAD^+^ was approximately 71.4-fold higher than that for NADH (Supplementary Table S2). *Sy*MDH exhibited substrate inhibition by NAD^+^ (**Figure 2B**), and the value of *K*_i_ was 14.5 mM (Supplementary Table S1).

**TABLE 1:** Kinetic parameters of *Sy*MDH.

	***V*_max_** (units·mg^–1^)	***k*_cat_** **(s^–1^)**	***k*_cat_/*K*_m_** **(s^–1^·mM^–1^)**
Malate	0.412	0.43	0.165
OAA	0.685	0.71	59.5
NAD^+^	0.199	0.21	0.357
NADH	0.931	0.97	32.3

*The oxidative reaction (malate to oxaloacetate) was assayed in 100 mM potassium phosphate buffer (pH 8.0) by varying the malate concentration at a fixed NAD^+^ concentration (8.0 mM) or by varying the NAD^+^ concentration at a fixed malate concentration (4.0 mM). The reductive reaction (oxaloacetate to malate) was assayed in 100 mM potassium phosphate buffer (pH 6.5) by varying the oxaloacetate concentration at a fixed NADH concentration (0.1 mM) or by varying the NADH concentration at a fixed oxaloacetate concentration (0.1 mM). The kinetic parameters were calculated by the Lineweaver–Burk plot. The values of k_*cat*_ were calculated by dividing V_*max*_ by the molar amounts of SyMDH proteins.*

**TABLE 2:** Comparison of *K*_m_-values of MDHs in various microorganisms.

***K*_m_ (μM)**	**Malate**	**OAA**	**NAD^+^**	**NADH**	**Malate/OAA**	**NAD^+^/NADH**	**Reference**
*Nitrosomonas europaea*	5000	20	24	22	250	1.1	Deutch, 2013
*Synechocystis* sp. PCC 6803	2600	12	580	30	216.7	19.3	This study
Syntrophic propionate-oxidising *bacterium strain MPOB*	4000	50	1100	30	80	36.7	van Kuijk and Stams, 1996
*Methanobacterium thermoautotrophicum*	400	30	90	90	13.3	1	Thompson et al., 1997
*Bacillus subtilis* B1	260	22	100	14	11.8	7.1	Wynne et al., 1996
*Haemophilus parasuis*	550	72	120	17	7.6	7.1	Wise et al., 1997
*Streptomyces coelicolor*	490	190	150	83	2.6	1.8	Ge et al., 2010
*Pseudomonas stutzeri*	63	32	340	36	2	9.4	Labrou and Clonis, 1997
*Helicobacter pylori*	180	130	160	65	1.4	2.5	Pitson et al., 1999
*Methanothermus fervidus*	150	200	140	5	0.8	28	Honka et al., 1990

**K_*m*_-values are listed in ascending order of K_*m*_ for malate/K_*m*_ for oxaloacetate. K_*m*_-values were calculated by the Lineweaver–Burk plot*.*

### Effect of Various Effectors on *Sy*MDH Activity

**FIGURE 4:**
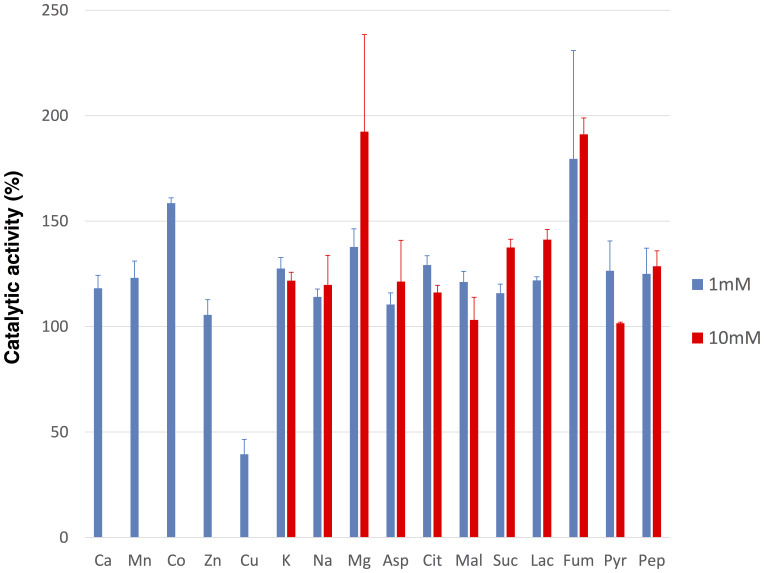
Effects of various metal ions and compounds on the *Sy*MDH in the reductive reaction *in vitro*. 10 μg of *Sy*MDH was pre-incubated with 100 mM potassium phosphate (pH 6.5), 0.1 mM NADH, 0.1 mM oxaloacetate and effectors, at 45°C. The graphs show the mean ± SD obtained from three independent experiments. Activity of *Sy*MDH in the absence of effectors was set at 100%. Ca, CaCl_2_; Mn, MnCl_2_⋅4H_2_O; Co, Co(NO_3_)_2_⋅6H_2_O; Zn, ZnSO_4_⋅7H_2_O; Cu, CuSO_4_⋅5H_2_O; K, KCl; Na, NaCl; Mg, MgCl_2_; Asp, L-Aspartate; Cit, Citrate; Mal, L-Malate; Suc, Succinate; Lac, L-lactate; Fum, Fumarate; Pyr, Pyruvate; Pep, Phosphoenolpyruvate.

**FIGURE 5:**
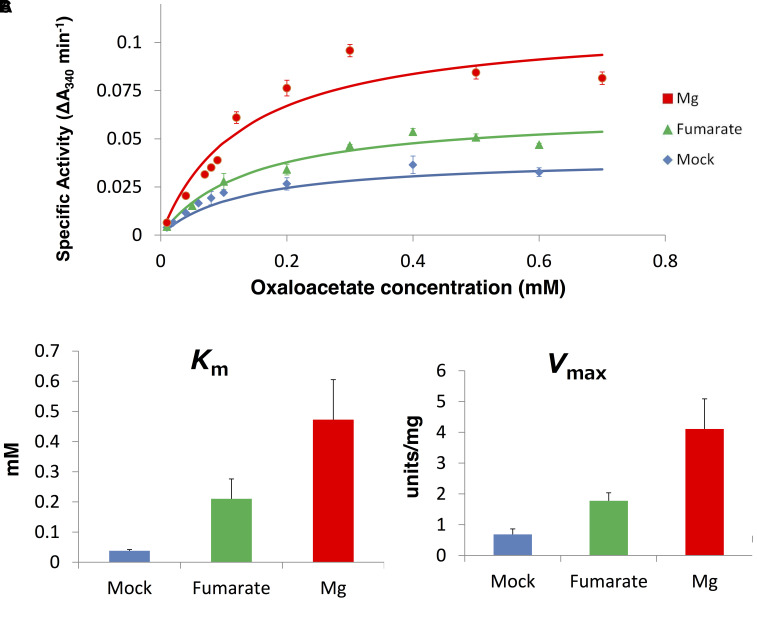
The *K*_m_ and *V*_max_-values for oxaloacetate in the presence of 10 mM fumarate and 10 mM magnesium ion *in vitro*. **(A)** Saturation curves of the activity of *Sy*MDH. Blue line indicates mock, green line indicates presence of fumarate, and red line indicates the presence of magnesium. The graph shows the mean of three independent experiments. **(B)** *K*_m_ (mean ± SD) (units/mg protein) values in the presence of 10 mM fumarate and 10 mM magnesium ion, obtained from three independent experiments. **(C)** *V*_max_ (mean ± SD) values for oxaloacetate, obtained from three independent experiments. Mock indicates the enzymatic activity in the absence of additional compounds.

The reductive reaction catalysed by bacterial MDHs is inhibited by TCA cycle metabolites, such as excess oxaloacetate and divalent metal ions (Takahashi-Íñiguez et al., 2016). Therefore, we measured the activity of *Sy*MDH in the reductive reaction in the presence of various effectors. *Sy*MDH was inhibited by excess NAD^+^ in the reductive reaction (**Figure 2B**). With the exception of cobalt, magnesium, and copper ions, all other metal ions showed little effect on *Sy*MDH (**Figure 4**). *Sy*MDH activity increased approximately 140 and 160% with the addition of 1 mM Co(NO_3_)_2_⋅6H_2_O and 1 mM MgCl_2_, respectively (**Figure 4**). In the presence of 10 mM MgCl_2_, the activity of *Sy*MDH increased to approximately 190% (**Figure 4**). Among the metal ions tested, only copper ions reduced the activity of *Sy*MDH. In the presence of 1 mM CuSO_4_⋅5H_2_O, *Sy*MDH activity decreased to approximately 40% of normal activity (**Figure 4**). *Sy*MDH activity could not be measured in the presence of 10 mM calcium, manganese, cobalt, zinc, or copper ions due to the formation of a precipitate (**Figure 4**). *Sy*MDH activity rose approximately 170 and 190% with the addition of 1 and 10 mM fumarate, respectively (**Figure 4**). *Sy*MDH activity with oxaloacetate at a concentration of 0.01–0.6 mM was measured in the presence of 10 mM magnesium and fumarate, and the kinetic parameters were calculated by Lineweaver–Burk plots (**Figure 5A**). Both the *K*_m_ and *V*_max_-values of this substrate and reaction, respectively, increased with the addition of 10 mM MgCl_2_ and fumarate (**Figures 5B,C**). To strengthen the validity of our results, we also performed biochemical assays using cell extracts (Supplementary Figure S3a). Unlike *in vitro*, the *K*_m_-value did not change *in vivo* in the presence of 10 mM MgCl_2_ and fumarate (Supplementary Figure S3b). The *V*_max_-value increased *in vivo* similar to *in vitro* in the presence of 10 mM MgCl_2_ and fumarate (Supplementary Figure S3c).

### Thermal Properties of *Sy*MDH Activity

**FIGURE 6:**
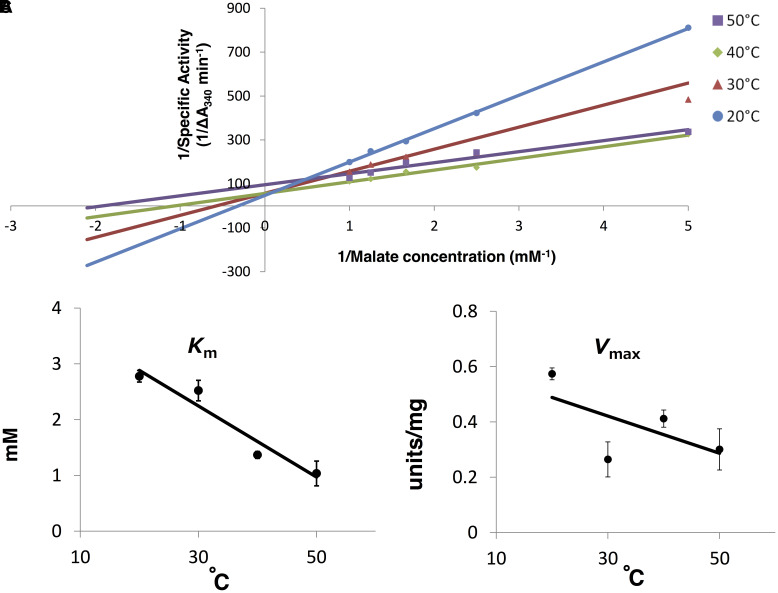
Thermal profiles of *Sy*MDH in the oxidative reaction *in vitro*. **(A)** Lineweaver–Burk plot of the *Sy*MDH activity in the oxidative reaction at 20–50°C. Blue, red, green, and purple lines indicate condition at 20, 30, 40, and 50°C, respectively. The graph shows the mean of three independent experiments. **(B)** *K*_m_ (mean ± SD) values for malate were obtained from three independent experiments by varying the temperature (20–50°C). **(C)** *V*_max_ (mean ± SD) (units/mg protein) values for malate were obtained from three independent experiments by varying the temperature (20–50°C).

**FIGURE 7:**
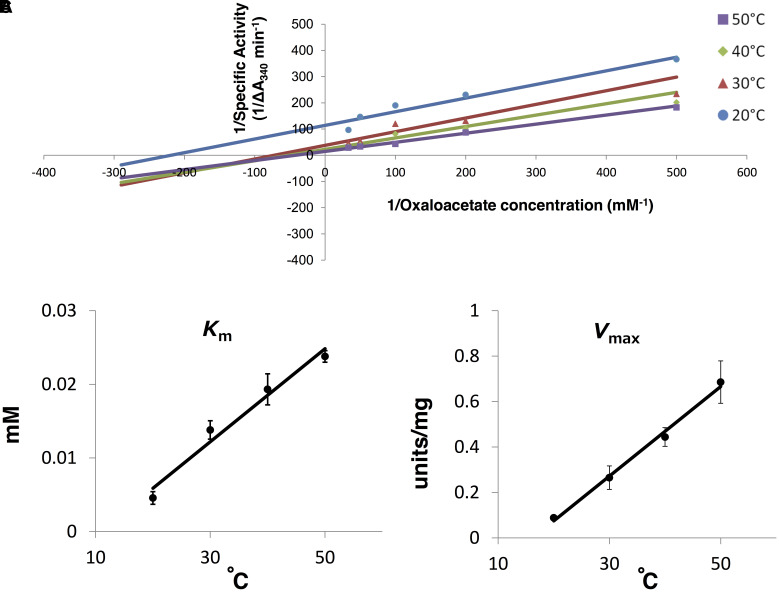
Thermal profiles of *Sy*MDH in the reductive reaction *in vitro*. **(A)** Lineweaver–Burk plot of the *Sy*MDH activity in the reductive reaction at 20–50°C. Blue, red, green, and purple lines indicate condition at 20, 30, 40, and 50°C, respectively. The graph shows the mean of three independent experiments. **(B)** *K*_m_ (mean ± SD) values for oxaloacetate were obtained from three independent experiments by varying the temperature (20–50°C). **(C)** *V*_max_ (mean ± SD) (units/mg protein) values for oxaloacetate were obtained from three independent experiments by varying the temperature (20–50°C).

*Synechocystis* 6803 MDH activity was measured by varying temperature (20–50°C). The *K*_m_ and the *V*_max_ were calculated by both a Lineweaver–Burk double reciprocal plot (**Figures 5**, **6**) and curve fitting used the Michaelis–Menten equation (Supplementary Figures S4, S5). The *K*_m_ and the *V*_max_-values for malate tend to decrease as the temperature rise, although the *V*_max_-values less dependent on the temperature (**Figure 6** and Supplementary Figure S4). On the contrary, the *K*_m_ and the *V*_max_-values for oxaloacetate increased as the temperature rise (**Figure 7** and Supplementary Figure S5). The *K*_m_ and the *V*_max_ for malate at 20°C were approximately 2.7-fold and 1.9-fold higher than that at 50°C, respectively (**Figure 6**). The *K*_m_ and *V*_max_ for oxaloacetate at 20°C were approximately 0.19- and 0.13-fold higher than that at 50°C, respectively (**Figure 7**). The *K*_m_ and *V*_max_ of *Sy*MDH demonstrated its temperature dependency.

## Discussion

We purified recombinant *Sy*MDH protein and revealed the biochemical properties of cyanobacterial MDH for the first time. The optimal pH of *Sy*MDH was different for the oxidative reaction and the reductive reaction (**Figure 1B**). Cyanobacteria utilise a reductive branch of TCA cycle and excrete succinate under dark anaerobic conditions (Hasunuma et al., 2016). The intracellular pH of cyanobacteria decreases during the transition from light to dark conditions (Coleman and Coleman, 1981; Mangan et al., 2016). Therefore, to adapt to the drastic changes in primary metabolism during the light and dark cycle, *Sy*MDH is thought to shift its substrate affinity according to the intracellular pH. *Sy*MDH was stable at a wide range of temperature, being particularly tolerant to high temperatures (**Figure 1C**). Among the mesophilic microorganisms, MDHs from *Streptomyces avermitilis*, *Streptomyces coelicolor*, and *Nitrosomonas europaea* maintain their activity at 50°C (Mikulášová et al., 1998; Ge et al., 2010; Deutch, 2013), but these MDHs are completely inactivated at 60–70°C (Mikulášová et al., 1998; Ge et al., 2010; Deutch, 2013). *Sy*MDH maintains its activity in both oxidative and reductive reactions at 60–70°C (**Figure 1C**). Therefore, *Sy*MDH is the most thermostable enzyme among MDHs from the mesophilic microorganisms investigated thus far. The optimal temperatures of *Sy*MDH were 50 and 45°C, for the oxidative and reductive reaction, respectively (**Figure 1C**). Thus, optimal temperature of *Sy*MDH (45–50°C) and optimal growth temperature of *Synechocystis* 6803 (30–35°C) were different. Generally, an enzymatic reaction is promoted by increasing temperature, because the kinetic energy of the reactants increases. However, an enzyme denature at high temperatures. Since *Sy*MDH is a heat-stable enzyme (**Figure 1B**), the enzyme activity became the highest at around 50°C, which is higher than the optimal growth temperature in *Synechocystis* 6803. Besides *Synechocystis* 6803, microorganisms having the MDHs with the optimal temperature much higher than the optimal growth temperature are *S. avermitilis* and *S. coelicolor*, *N. europaea* (Mikulášová et al., 1998; Ge et al., 2010; Deutch, 2013). *Sy*MDH activity was suppressed by copper (**Figure 4**), as was observed for the MDH from *Pseudomonas stutzeri* (Labrou and Clonis, 1997). *P. stutzeri* MDH is also inhibited by citrate (Labrou and Clonis, 1997), but *Sy*MDH was slightly activated by citrate (**Figure 4**). The only reported activators of bacterial MDHs are >0.18 mM malate and 3 M NaCl (Cendrin et al., 1997; Labrou and Clonis, 1997), but *Sy*MDH was significantly activated by magnesium ions and fumarate (**Figure 5** and Supplementary Figure S3), suggesting that *Sy*MDH is regulated by a positive feedback mechanism. These results are indicative of the diversity of regulation among MDHs. Intracellular concentrations of malate and fumarate in *E. coli* cells are 1.7 and 0.11 mM, respectively (Bennett et al., 2009). Since *Sy*MDH showed maximum activity at 5 mM malate (**Figure 2**) and was activated with 1 mM fumarate (**Figure 4**), it is plausible that *Sy*MDH activity was regulated by the TCA cycle metabolites. Excess NAD^+^ (>4 mM) caused substrate inhibition in *Sy*MDH (**Figure 2B**). MDHs from *Methanobacterium thermoautotrophicum* and *P. stutzeri* are also inhibited by excess NAD^+^ (>0.5 mM) and NAD^+^ (>250 mM), respectively (Labrou and Clonis, 1997; Thompson et al., 1997). Intracellular concentrations of NAD^+^ in *E. coli* cells is 2.6 mM (Bennett et al., 2009), thus, *Sy*MDH activity is thought to inhibited by NAD^+^ present in *Synechocystis* 6803.

The affinity of oxaloacetate and NADH for *Sy*MDH was higher than the affinity of malate and NAD^+^, respectively (**Table 1**). Generally, bacterial MDHs show higher affinity for oxaloacetate than malate (Takahashi-Íñiguez et al., 2016), and *Sy*MDH was consistent with this. When comparing the substrate affinity among bacterial MDHs, the *K*_m_ (malate)/*K*_m_ (oxaloacetate) ratio in descending order is as follows: *N. europaea* (250)*, Synechocystis* 6803 (210), Syntrophic propionate-oxidising bacterium strain MPOB (80.0), and *Methanobacterium thermoautotrophicum* (13.3) (**Table 2**). The previous study demonstrated that the NAD^+^ concentration is approximately 500 times higher than NADH concentration in *Synechocystis* 6803 (Osanai et al., 2014). Therefore, although our biochemical analysis showed that *Sy*MDH has higher coenzyme specificity toward NADH than NAD^+^, *Sy*MDH can catalyse both reductive and oxidative reactions *in vivo*. The *K*_m_ (NAD^+^)/*K*_m_ (NADH) ratio in descending order is as follows: Syntrophic propionate-oxidising bacterium strain MPOB (36.7), *Methanothermus fervidus* (28.0), and *Synechocystis* 6803 (19.0). These aforementioned microorganisms are thought to have low MDH activity in the oxidation reaction. This is because *N. europaea* is deficient in 2-oxoglutarate dehydrogenase (Beyer et al., 2009) and succinyl-CoA is formed via phosphoenolpyruvate and oxaloacetate using a reductive branch of TCA cycle (Deutch, 2013). In addition, syntrophic propionate-oxidising bacterium strain MPOB, *Methanobacterium thermoautotrophicum*, and *Methanothermus fervidus* are anaerobic microorganisms (Harmsen et al., 1996; Thompson et al., 1997; Stetter et al., 1981), and therefore, their oxidative TCA cycles are barely functioning. As with microorganisms in which the oxidative TCA cycle does not appear to function, the *K*_m_ (for malate)/*K*_m_ (for oxaloacetate) ratio and the *K*_m_ (NAD^+^)/*K*_m_ (NADH) ratio of *Sy*MDH were very high. Therefore, *Sy*MDH is likely to have low activity in the oxidative reaction. This conclusion is supported by flux analyses. Previous studies measured metabolic flow by estimating the flux rates of metabolites per dry cell weight (DCW) per unit hour in *Synechocystis* 6803 under mixotrophic conditions and found that all fluxes in TCA cycle reactions were clockwise (0.02–0.11 mmol gDCW^-1^ h^-1^), except for the interconversion between malate and oxaloacetate, which was anticlockwise (0.13 mmol gDCW^-1^ h^-1^; Nakajima et al., 2014). Similar results were observed under photoheterotrophic, nitrogen-limited, and dark conditions (Nakajima et al., 2014, 2017; Wan et al., 2017). *In vivo* studies have shown that many genes of the cyanobacterial TCA cycle are unnecessary for normal growth (Broddrick et al., 2016). Even if expression of fumarase, which catalyses the reversible hydration/dehydration of fumarate to malate, is blocked, growth of cyanobacteria under continuous light is not affected (Rubin et al., 2015). Therefore, the oxidative reaction of *Sy*MDH is also thought to be unnecessary in cyanobacteria, because fumarase-deficient cyanobacteria grow normally. These studies support our biochemical studies suggesting that the oxidative reaction of *Sy*MDH is very weak and almost non-functional. The kinetic parameters of *Sy*MDH were affected by temperature (**Figures 6**, **7** and Supplementary Figures S4, S5). *K*_m_-value for oxaloacetate was always lower than that for malate in range of 20–50°C, thus, it is considered that *Sy*MDH always show higher affinity for oxaloacetate than malate within 20–50°C and the reaction direction of *Sy*MDH tends to flow from oxaloacetate to malate within the growth temperature of *Synechocystis* 6803.

Our study revealed that *Sy*MDH shows a higher affinity for substances produced through the reductive reaction than those produced through the oxidative reaction, similar to MDHs derived from anaerobic microorganisms in which the oxidative TCA cycle seems to be barely functioning. Cyanobacteria have been found to close the TCA cycle using various bypasses (Zhang and Bryant, 2011; Steinhauser et al., 2012; Xiong et al., 2014). However, the results in this study indicate that the oxidative TCA cycle of *Synechocystis* 6803 may be functionally linear, and not cyclic in nature, because *Sy*MDH preferentially undergoes a reductive reaction rather than an oxidative reaction and turns off the cyclic process of the oxidative TCA cycle.

## Author Contributions

MT designed the research, performed the experiments, analysed the data, and wrote the manuscript. SI analysed the data. HS performed the experiments. TO analysed the data and wrote the manuscript.

## Conflict of Interest Statement

The authors declare that the research was conducted in the absence of any commercial or financial relationships that could be construed as a potential conflict of interest.
