# Corrigendum: Purification and Characterisation of Malate Dehydrogenase From *Synechocystis* sp. PCC 6803: Biochemical Barrier of the Oxidative Tricarboxylic Acid Cycle

**DOI:** 10.3389/fpls.2021.699371

**Published:** 2021-06-11

**Authors:** Masahiro Takeya, Shoki Ito, Haruna Sukigara, Takashi Osanai

**Affiliations:** School of Agriculture, Meiji University, Tokyo, Japan

**Keywords:** biochemistry, cyanobacteria, malate dehydrogenase, metabolic enzyme, TCA cycle

In the original article, there were errors in Table 1 and Table S1 as published as a result of the incorrect calculation of *k*_cat_. We performed a re-calculation of and *k*_cat_ values and corrected the *k*_cat_ and *k*_cat_/*K*_*m*_ values in Table 1 and Table S1; the corrected versions these tables and updated Table 1 footnote appear below.

**Table 1:** Kinetic parameters of *Sy*MDH.

	***V*_**max**_**	***k*_**cat**_**	***k*_**cat**_/*K*_**m**_**
	**(units·mg^**−1**^)**	**(s^**−1**^)**	**(s^**−1**^·mM^**−1**^)**
Malate	0.412	0.43	0.165
OAA	0.685	0.71	59.5
NAD^+^	0.199	0.21	0.357
NADH	0.931	0.97	32.3

*The oxidative reaction (malate to oxaloacetate) was assayed in 100 mM potassium phosphate buffer (pH 8.0) by varying the malate concentration at a fixed NAD^+^ concentration (8.0 mM) or by varying the NAD^+^ concentration at a fixed malate concentration (4.0 mM). The reductive reaction (oxaloacetate to malate) was assayed in 100 mM potassium phosphate buffer (pH 6.5) by varying the oxaloacetate concentration at a fixed NADH concentration (0.1 mM) or by varying the NADH concentration at a fixed oxaloacetate concentration (0.1 mM). The kinetic parameters were calculated by the Lineweaver–Burk plot. The values of k_cat_ were calculated by dividing V_max_ by the molar amounts of SyMDH proteins*.

**Table S1:** Kinetic parameters of *Sy*MDH calculated by Michaelis-Menten equation.

	***V*_**max**_**	***k*_**cat**_**	***k*_**cat**_/*K*_**m**_**	***K_**i**_***
	**(units·mg^**−1**^)**	**(s^**−1**^)**	**(s^**−1**^·mM^**−1**^)**	**(mM)**
Malate	0.373	0.39	0.144	–
OAA	0.422	0.44	13.7	–
NAD^+^	0.647	0.67	0.674	14.5
NADH	1.795	1.87	134	–

*The oxidative reaction (malate to oxaloacetate) was assayed in 100 mM potassium phosphate buffer (pH 8.0) by varying the malate concentration at a fixed NAD^+^ concentration (8.0 mM) or by varying the NAD^+^ concentration at a fixed malate concentration (4.0 mM). The reductive reaction (oxaloacetate to malate) was assayed in 100 mM potassium phosphate buffer (pH 6.5) by varying the oxaloacetate concentration at a fixed NADH concentration (0.1 mM) or by varying the NADH concentration at a fixed oxaloacetate concentration (0.1 mM). The kinetics parameters were calculated by the Michaelis-Menten equation*.

The following sentence in the *Results* sub-section the *Measurement of Kinetic Parameters* contained an associated error: “*Sy*MDH displayed approximately 1.7-fold (*k*_cat_) and 350-fold (*k*_cat_/*K*_m_) preferences for oxaloacetate reduction over malate oxidation and approximately 4.7-fold (*k*_cat_) and 89.5-fold (*k*_cat_/*K*_m_) preferences for NADH oxidation over NAD^+^ reduction (Table 1).” This sentence should have read: “*Sy*MDH displayed approximately 1.7-fold (*k*_cat_) and 361-fold (*k*_cat_/*K*_*m*_) preferences for oxaloacetate reduction over malate oxidation and approximately 4.7-fold (*k*_cat_) and 90.5-fold (*k*_cat_/*K*_*m*_) preferences for NADH oxidation over NAD^+^ reduction (Table 1).”

The authors apologize for this error and state that this does not change the scientific conclusions of the article in any way. The original article has been updated.

