# Supplementary material for: Purification and Characterisation of Malate Dehydrogenase From Synechocystis sp. PCC 6803: Biochemical Barrier of the Oxidative Tricarboxylic Acid Cycle
Source: Front Plant Sci. 2018 Jul 13;9:947. doi: 10.3389/fpls.2018.00947 (PMC6053527; doi:10.3389/fpls.2018.00947)
Supplement: Supplementary file 1 [file Table_1.DOCX]

Table S1. Kinetic parameters of *Sy*MDH calculated by Michaelis-Menten equation

|  | *V*_max_ | *k*_cat_ | *k*_cat_/*K*_m_ | *K_i_* |
| --- | --- | --- | --- | --- |
|  | (units・mg^-1^) | (S^-1^) | (S^-1^・mM^-1^) | (mM) |
| Malate | 0.373 | 0.39 | 0.144 | - |
| OAA | 0.422 | 0.44 | 13.7 | - |
| NAD^+^ | 0.647 | 0.67 | 0.674 | 14.5 |
| NADH | 1.795 | 1.87 | 134 | - |

The oxidative reaction (malate to oxaloacetate) was assayed in 100 mM potassium phosphate buffer (pH 8.0) by varying the malate concentration at a fixed NAD^+^ concentration (8.0 mM) or by varying the NAD^+^ concentration at a fixed malate concentration (4.0 mM). The reductive reaction (oxaloacetate to malate) was assayed in 100 mM potassium phosphate buffer (pH 6.5) by varying the oxaloacetate concentration at a fixed NADH concentration (0.1 mM) or by varying the NADH concentration at a fixed oxaloacetate concentration (0.1 mM). The kinetics parameters were calculated by the Michaelis-Menten equation.
