# Supplementary material for: Purification and Characterisation of Malate Dehydrogenase From Synechocystis sp. PCC 6803: Biochemical Barrier of the Oxidative Tricarboxylic Acid Cycle
Source: Front Plant Sci. 2018 Jul 13;9:947. doi: 10.3389/fpls.2018.00947 (PMC6053527; doi:10.3389/fpls.2018.00947)
Supplement: Supplementary file 2 [file Table_2.DOCX]

Table S2. *K*_m_ values of *Sy*MDH calculated by the Michaelis-Menten equation

| *K*_m_ (μM) | Malate | OAA | NAD^+^ | NADH | Malate/OAA | NAD^+^/NADH |
| --- | --- | --- | --- | --- | --- | --- |
|  | 2700 | 32 | 1000 | 14 | 84.4 | 71.4 |

*K*_m_ values of *Sy*MDH were determined by the Michaelis-Menten equation using the specific activity in figures 2 and 3.
