# Supplementary material for: Purification and Characterisation of Malate Dehydrogenase From Synechocystis sp. PCC 6803: Biochemical Barrier of the Oxidative Tricarboxylic Acid Cycle
Source: Front Plant Sci. 2018 Jul 13;9:947. doi: 10.3389/fpls.2018.00947 (PMC6053527; doi:10.3389/fpls.2018.00947)
Supplement: Supplementary file 3 [file Data_Sheet_1.docx]

Supplementary Figure 1. Measurement of *Sy*MDH activity toward NADP^+^ and NADPH *in vitro*. (a) Activity was measured by varying the NADP^+^ concentration at a fixed malate concentration (4.0 mM) using 10 μg of *Sy*MDH. The graphs show the mean ± SD obtained from three independent experiments. (b) Activity was measured by varying the NADPH concentration at a fixed oxaloacetate concentration (0.1 mM) using 10 μg of *Sy*MDH. The graphs show the mean ± SD obtained from three independent experiments.

Supplementary Figure 2. Measurement of *Sy*MDH activity toward NADP^+^ and NADPH *in vivo*. (a) Activity was measured by varying the NADP^+^ concentration at a fixed malate concentration (4.0 mM) using 420 μg of total proteins. The graphs show the mean ± SD obtained from three independent experiments. (b) Activity was measured by varying the NADPH concentration at a fixed oxaloacetate concentration (0.1 mM) using 420 μg of total proteins. The graphs show the mean ± SD obtained from three independent experiments.

Supplementary Figure 3. The *K*_m_ and *V*_max_ values for oxaloacetate in the presence of 10 mM fumarate and 10 mM magnesium ion *in vivo*. (a) Saturation curves of the activity of *Sy*MDH. Blue line indicates mock, green line indicates presence of fumarate and red line indicates the presence of magnesium. The graph shows the mean of three independent experiments. (b) *V*_max_ (mean ± SD) (units/mg protein) values in the presence of 10 mM fumarate and 10 mM magnesium ion, obtained from three independent experiments. (c) *K*_m_ (mean ± SD) values for oxaloacetate, obtained from three independent experiments. Mock indicates the enzymatic activity in the absence of additional compounds.

Supplementary Figure 4. Thermal profiles of *Sy*MDH in the oxidative reaction *in vitro*. (a) Saturation curves of the *Sy*MDH activity in the oxidative reaction at 20-50°C. Blue, red, green, and purple lines indicate condition at 20, 30, 40, and 50°C, respectively. The graph shows the mean of three independent experiments. (b) *K*_m_ (mean ± SD) values for malate calculated by the Michaelis-Menten equation were obtained from three independent experiments by varying the temperature (20-50°C). (c) *V*_max_ (mean ± SD) (units/mg protein) values for malate calculated by the Michaelis-Menten equation were obtained from three independent experiments by varying the temperature (20-50°C).

Supplementary Figure 5. Thermal profiles of *Sy*MDH in the reductive reaction *in vitro*. (a) Saturation curves of the *Sy*MDH activity in the reductive reaction at 20-50°C. Blue, red, green, and purple lines indicate condition at 20, 30, 40, and 50°C, respectively. The graph shows the mean of three independent experiments. (b) *K*_m_ (mean ± SD) values for oxaloacetate calculated by the Michaelis-Menten equation were obtained from three independent experiments by varying the temperature (20-50°C). (c) *V*_max_ (mean ± SD) (units/mg protein) values for oxaloacetate calculated by the Michaelis-Menten equation were obtained from three independent experiments by varying the temperature (20-50°C).
