# Supplementary figures and images for: Purification and Characterisation of Malate Dehydrogenase From Synechocystis sp. PCC 6803: Biochemical Barrier of the Oxidative Tricarboxylic Acid Cycle
Source: Front Plant Sci. 2018 Jul 13;9:947. doi: 10.3389/fpls.2018.00947 (PMC6053527; doi:10.3389/fpls.2018.00947)

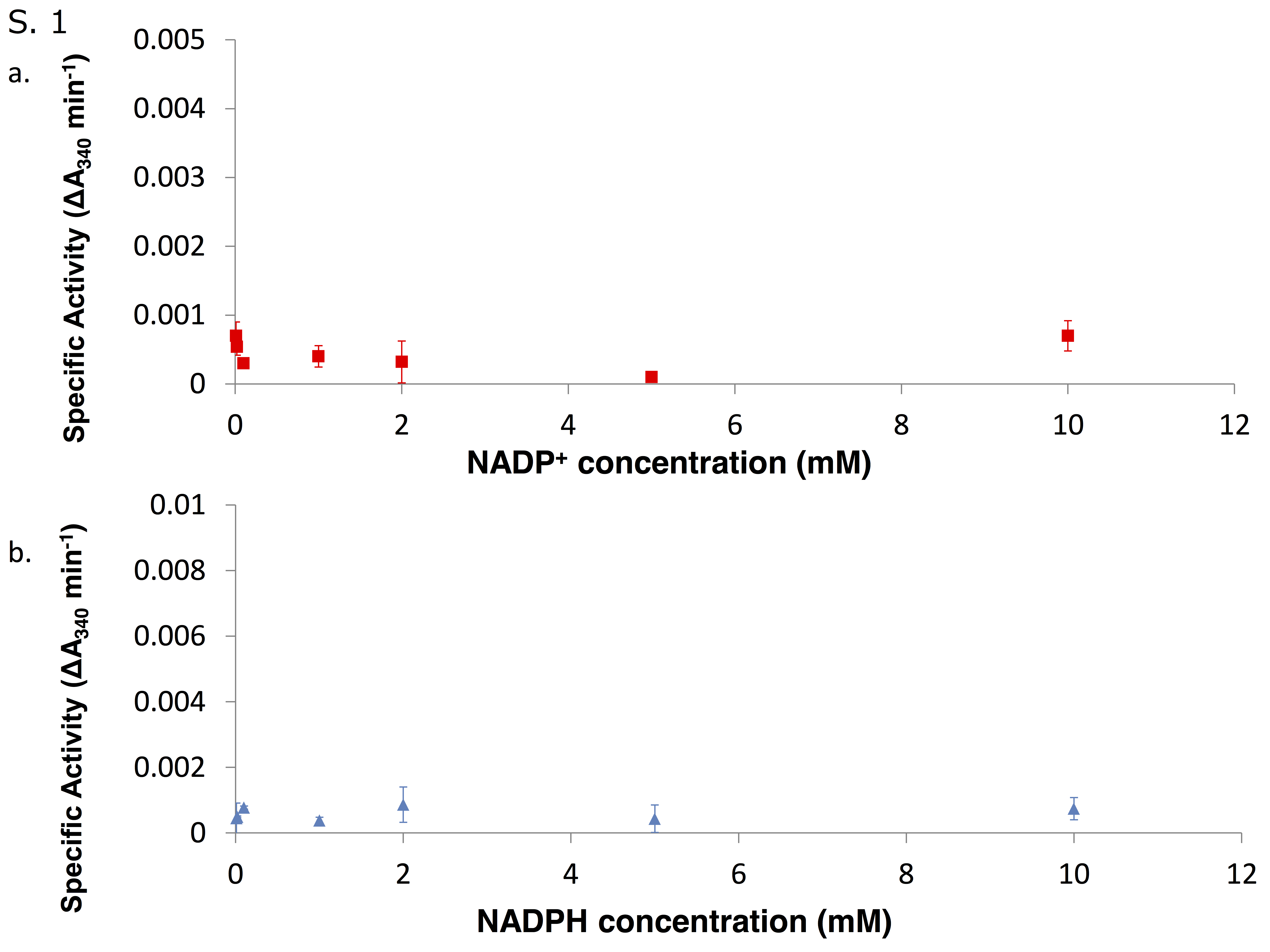

Supplement: Supplementary file 4 [file Image_1.TIFF]

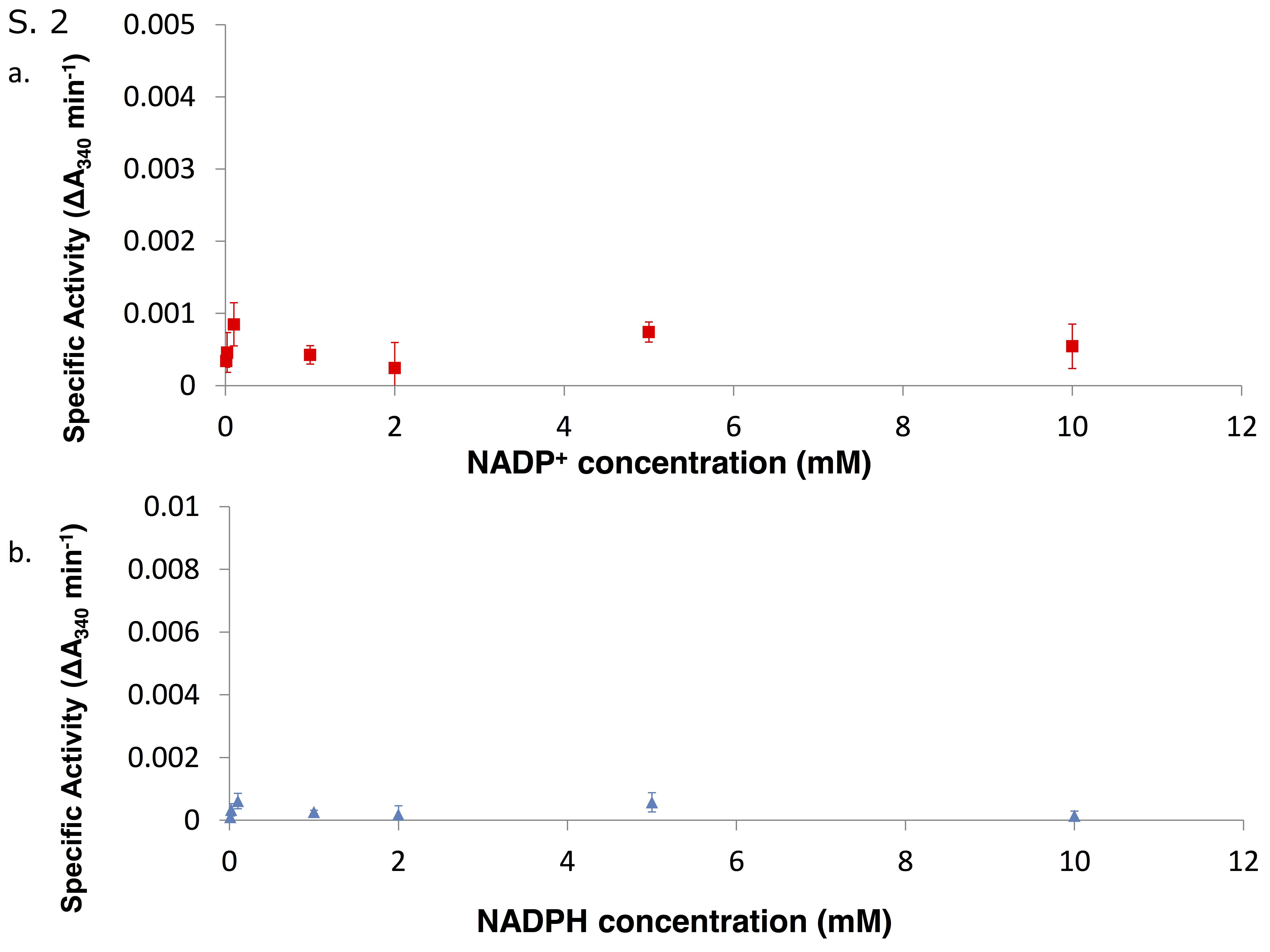

Supplement: Supplementary file 5 [file Image_2.TIFF]

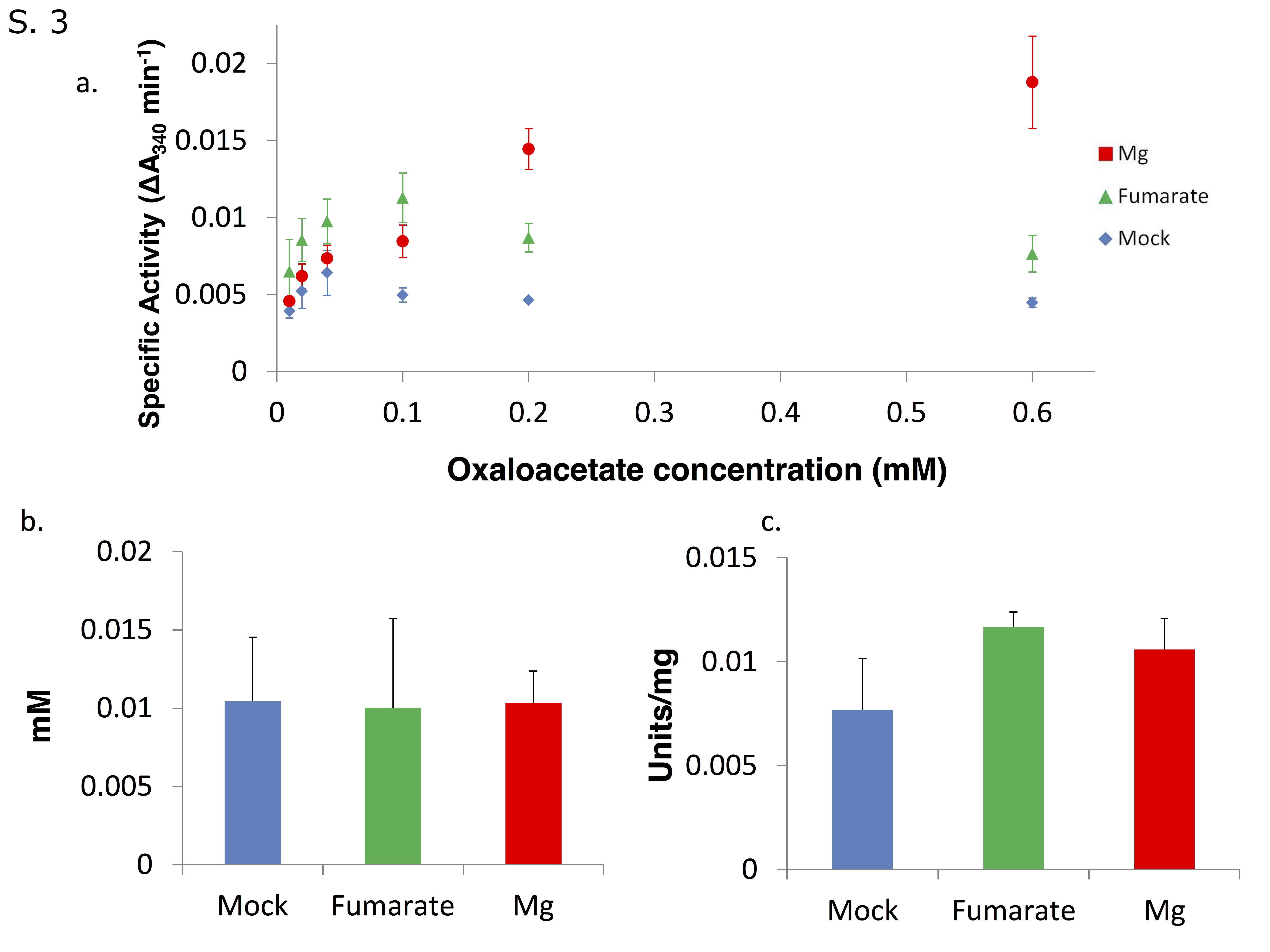

Supplement: Supplementary file 6 [file Image_3.TIFF]

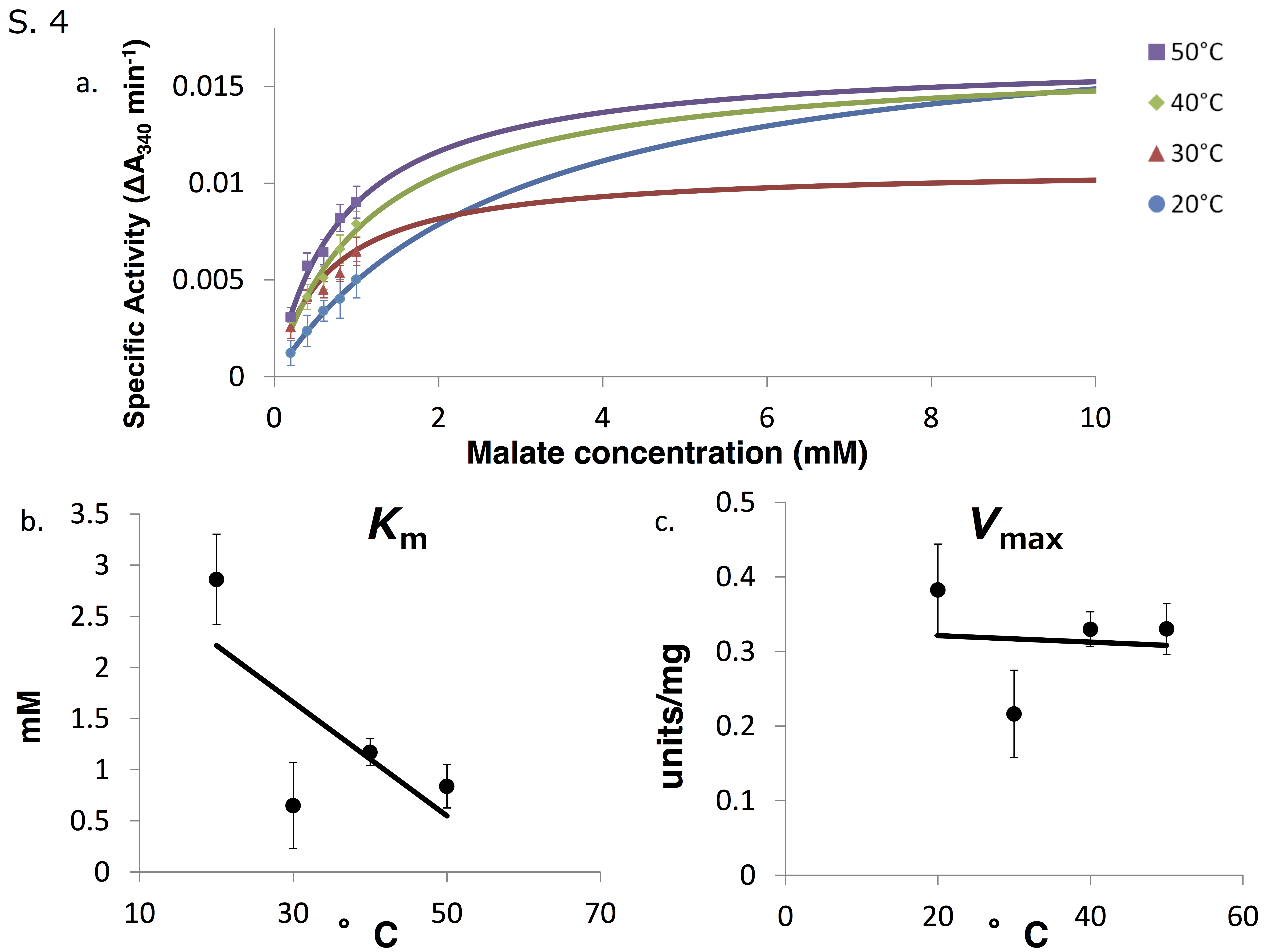

Supplement: Supplementary file 7 [file Image_4.TIFF]

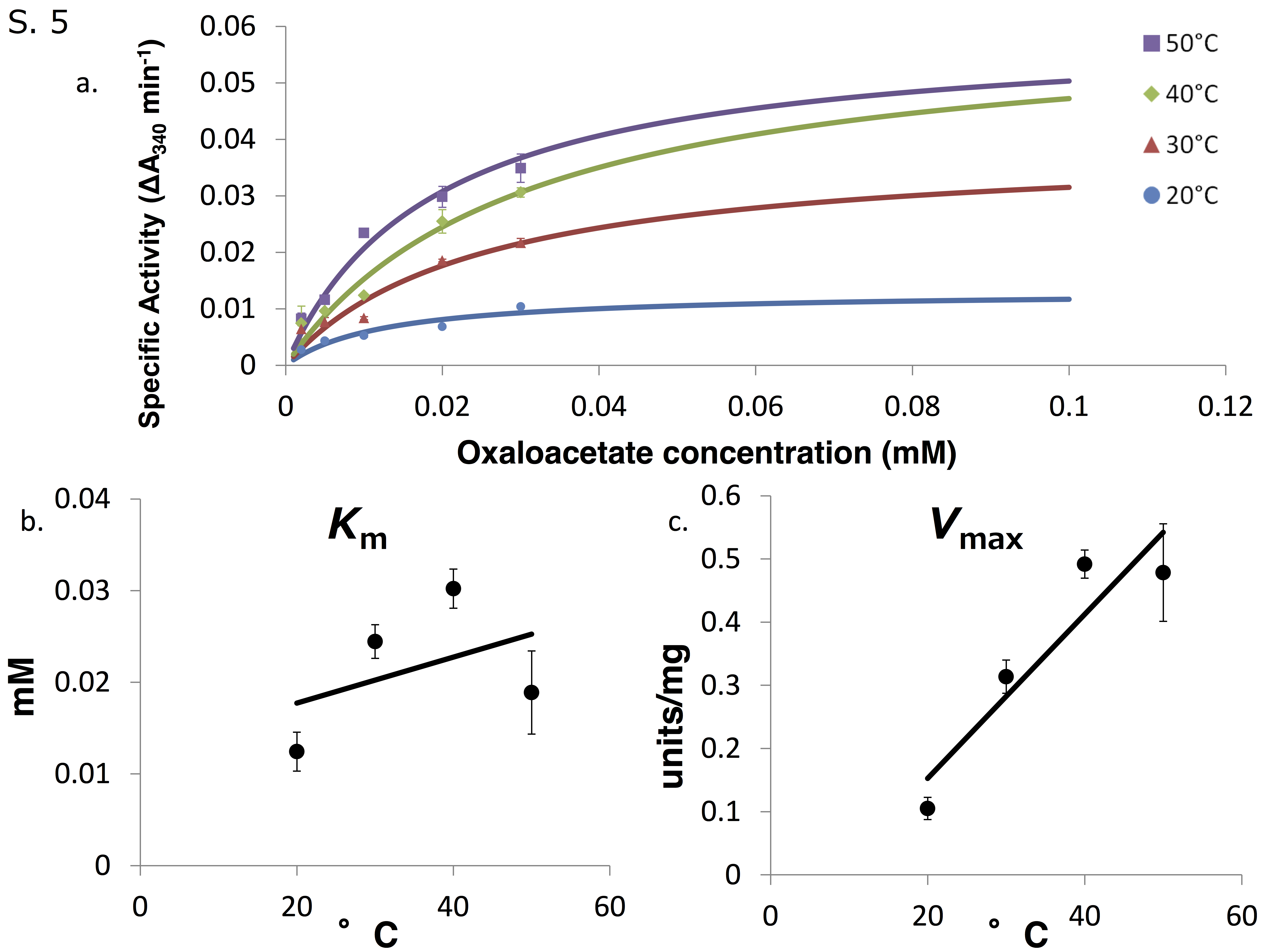

Supplement: Supplementary file 8 [file Image_5.TIFF]
